# Complete Genome Sequence of the *Arcobacter butzleri* Cattle Isolate 7h1h

**DOI:** 10.1128/genomeA.00655-13

**Published:** 2013-08-22

**Authors:** J. Yvette Merga, Craig Winstanley, Nicola J. Williams, Emma Yee, William G. Miller

**Affiliations:** Institute of Infection and Global Health, University of Liverpool, Liverpool, United Kingdoma; Produce Safety and Microbiology Research Unit, U.S. Department of Agriculture, Albany, California, USAb

## Abstract

*Arcobacter butzleri* strain 7h1h was isolated in the United Kingdom from the feces of a clinically healthy dairy cow. The genome of this isolate was sequenced to completion. Here, we present the annotation and analysis of the completed 7h1h genome, along with a comparison of this genome to the existing *A. butzleri* genomes.

## GENOME ANNOUNCEMENT

*Arcobacter butzleri* is a member of the *Epsilonproteobacteria*, a taxonomic division that also contains the established pathogens *Campylobacter jejuni* and *Helicobacter pylori*. *A. butzleri* has been isolated from water, food animals, and multiple food sources (1). *A. butzleri* has also been associated with human gastroenteritis (1). *A. butzleri* strain 7h1h originated from a clinically healthy dairy cow in Cheshire, United Kingdom. It was assigned to a species using a previously used PCR (2) and was selected for sequencing based on the high quality of the amplicon that was produced. Multilocus sequence typing of the isolate (3) revealed it was a novel sequence type (ST-347), which was closely related to sequence type 303 (ST-303), isolated in the same study (with 6 allele matches). No other isolates shared >3 alleles with 7h1h. The complete genome sequences of two *A. butzleri* strains, the human clinical isolate RM4018 (4) and ED-1, isolated from a microbial fuel cell (5), were determined previously, and an incomplete 7h1h genome sequence was compared with that of RM4018 (6); here, we present the complete genome sequence of strain 7h1h.

Genome sequencing was performed using general and paired-end (8 to 12 kb) libraries and was generated on a Roche 454 FLX+ genome sequencer with Titanium chemistry. Newbler assembler (v2.6) was used to assemble 186,913 shotgun and 108,665 paired-end reads into a single scaffold of 27 contigs, providing 52× coverage. Scaffold gaps were filled using the 454 repeat contigs and the Perlscript Contig_extender3. Contig junctions were validated using amplification and Sanger sequencing. All 454 base calls were validated using 1,164,896 Illumina MiSeq reads, providing an additional 78× coverage.

The *A. butzleri* 7h1h genome size is 2,253,233 bp, with a G+C content of 27.06%. Protein-coding, rRNA-coding, and tRNA-coding genes were identified using GeneMark (v2.8; http://exon.gatech.edu/GeneMark/gmhmm2_prok.cgi), RNAmmer v1.2 (7), and tRNAscan-SE (8), respectively. The gene start points were curated using Artemis (9). Final annotation was performed by BLASTp comparison to the proteomes of RM4018 and/or ED-1 or to proteins in the NCBI nonredundant database, and by identification of Pfam domains (v.26.0 [10]). The 7h1h genome is predicted to carry 2,199 genes, 5 ribosomal RNA operons, and 54 tRNAs.

The 7h1h genome is highly syntenic to both the RM4018 and ED-1 genomes; no large-scale rearrangements were observed with respect to the other two genomes. Of the 2,199 genes carried by strain 7h1h, 1,946 (88%) were also identified in either RM4018 or ED-1. Of the remainder, 111 genes are either contained within an integrated element or are predicted to encode surface structure-associated proteins. Three integrated elements, bounded by 13- to 45-bp direct repeats and adjacent to tRNA-coding genes, were identified in the 7h1h genome. Unique to strain 7h1h are four toxin-antitoxin family gene pairs and two families of insertion sequences (11 insertion sequence [IS] elements in total) that are unrelated to the mobile element identified in strain ED-1. Also present in strain 7h1h are genes encoding ATP-independent (urease) and ATP-dependent (urea carboxylase/allophanate hydrolase [11]) urea degradation pathways.

### Nucleotide sequence accession number. 

The genome sequence of *A. butzleri* strain 7h1h has been deposited in GenBank under the accession no. CP006615.
